# Person to person transmission of pneumonia associated with the novel severe acute respiratory syndrome coronavirus 2 (SARS-CoV-2): a familial cluster analysis in North Central Nigeria

**DOI:** 10.11604/pamj.2020.37.99.23609

**Published:** 2020-09-28

**Authors:** Adamu Ishaku Akyala, Liman Muhammad, Emmanuel Akabe, Akinyinka Akinyoade, Emmanuel Agogo

**Affiliations:** 1 Department of Microbiology, Faculty of Natural and Applied Sciences, Nasarawa State University, Keffi, Nasarawa State, Nigeria,; 2 Department of Epidemiology, State Ministry of Health, Lafia, Nasarawa State, Nigeria,; 3 African Studies Centre Leiden, University of Leiden, Wassenaarseweg 52, Leiden 2333AK, The Netherlands,; 4 Resolve to Save Lives, Abuja, Nigeria

**Keywords:** COVID-19, transmission, pneumonia, viral shedding

## Abstract

The continued absence of viable vaccines, limited diagnostic tools, insufficient protocol for isolation period, and weak health care system in developing countries with Nigeria inclusive heightens the tension trailing the arrival of Novel SARS-CoV-2 that was officially declared a global health emergency by World Health Organization (WHO) in January 2020. In this context, this study assesses the adequacy and potency of treatment for pneumonia associated with the Novel SARS-CoV-2. Counting from 27^th^ February 2020, exponential rise in cases of SARS-CoV-2 has been recorded in Nigeria. Despite limited data on person-to-person transmission or nosocomial transmission, we report the epidemiological features of a familial cluster of 4 positive cases to SARS-CoV-2 in Nasarawa State, North Central Nigeria. This cluster presented with an unexplained pneumonia after having contact with a family member who died after manifesting symptoms of Novel SARS-CoV-2; the test came out positive after his demise. Real-time reverse transcriptase polymerase chain reaction (RT-PCR) tests for SARS-CoV-2 nucleic acid were performed using nasopharyngeal swabs (Novel Coronavirus PCR Fluorescence Diagnostic Kit, BioGerm Medical Biotechnology at the Nigeria Centre for Disease Control (NCDC) in Abuja. Nigeria. From March 10, 2020, we enrolled a family of four patients out of a family of 10 who were positive to novel coronavirus. Four family members (aged 36-43 years) all presented with fever, upper or lower respiratory tract symptoms, or diarrhea, or a combination of these 3-6 days after exposure. These conditions continued to manifest at the Federal Medical Center, Keffi in Nasarawa State, 3-7 days after symptom onset. Their nasopharyngeal or throat swabs of these 10 family members were taken and four returned positive to coronavirus, while six tested negative. The epidemiological evidence from our study on familial cluster analysis reveals possible transmission of Novel SARS-CoV-2 during the incubation period. Study outcomes underscore the importance of probing contact history of potentially infected individuals, for prompt identification to preventing further spread.

## Introduction

In late December, 2019, an epidemic of respiratory disease caused by severe acute respiratory syndrome coronavirus 2 (SARS-CoV-2) began in the city of Wuhan in China which spread to over 30 countries of the world [1]. In the last 25 years, notable highly infectious respiratory viruses with pandemic potentials has emerged and remerged. Notable of which is the influenza virus that issued a global alert in 1918, 1957, 1968, 2003 and 2019 causing severe acute respiratory diseases [2]. The Novel SARS-CoV-2 outbreak resulted in globally, as of 2: 33pm CEST, 17 May 2020, there have been 4,525,497 confirmed cases of COVID-19, including 307,395 deaths, reported to WHO with substantial economic impact. Since then several other viral respiratory pathogens have emerged including Middle East respiratory syndrome coronavirus (MERS-CoV), adenovirus-14, and virulent strains of influenza viruses. Soon after the discovery of SARS, new coronaviruses NL63 and HKU1 were identified [2,3]. The emergence of 35 different respiratory viruses underscores the epidemic potential and overall threat to global health security. Severity caused by Novel SARS-CoV-2 has been recognized as a global public health security threat [3].

Many African countries are not prepared for the Novel SARS-CoV-2 outbreak due to poor and weak healthcare system, poor surveillance and response system, as well as inadequate and overstretched health facilities and services. Martinez-Alvarez *et al*. [4] established higher risk of Novel SARS-CoV-2 importation from Europe to Africa than china importation, comparing rapid spread of the virus in selected sub-Sahara countries than in European countries. Some African countries have developed capacity to respond to the outbreak as at 11 May, 2020, a total of 13,814 confirmed cases and 747 deaths from Novel SARS-CoV-2 have been documented in Africa [5]. The first index case of case of Novel SARS-CoV-2 was reported in Nigeria on the 27^th^ February 2020, this led to some public health interventions in order to reduce importation and local transmission slowing down the epidemic trajectory. The Nigeria Government implemented series of interventions among which include: ban on international travels to 15 countries on 20^th^ March, 2020 with closure of all schools and Universities with minimize mass gathering.

One key variable for measuring transmissibility of infectious diseases is the active reproduction number (Ref), which is related to the basic reproduction number (R0). The basic reproduction number (R0) is the average number of secondary cases that arises when one primary case is introduced into an uninfected population [5]. It is called the active reproduction number, (Ref) when this value changes during an epidemic [6]. Travel has remained a major source of concern for the current Novel SARS-CoV-2 pandemic; therefore, early transmissibility of the disease in Nigeria was quantified using a sequential Bayesian method, adjusting for disease importation. While much attention has been focused on viral transmission dynamics and the spectrum of clinical illness, these aspects are not yet completely understood [7-9]. This report describes the epidemiological and clinical features of Novel SARS-CoV-2 among four members of a family following SARS-CoV-2 infection in Nasarawa State, Northern Nigeria. Owing to the unavailability of effective vaccines for the prevention of SARS-CoV-2, most preventive measures aim to reduce the risk of infection.

## Methods

On the 1^st^ May, 2020. A patient from a family unit presented himself to the Federal Medical Center Keffi, in Nasarawa State, North Central Nigeria with pulmonary infiltrates, respiratory symptoms and fever. His symptoms fit into the case definition guideline of the Nigeria Center for Disease Control (NCDC). Preadmission evaluation also reveals his body temperature of 42°C, full blood count of 11.2 cells per liter and calcification of the aorta and aortic wall with pulmonary hypertension of both lungs. He has negative results for influenza A and B which was negative but had hyperglycemia with fasting blood sugar (FBS) of 23 milligrams per deciliter (mg/dL). Interstitial hyperplasia was reveal by computer tomography with infection in the left lungs posterior upper lobe segment. His throat and Nasopharyngeal swab were taken and placed in a viral medium for testing of Novel SARS-CoV-2 by Real Time PCR. On the 2^nd^ Junes, 2020 by 10:03 am the patient died and PCR results returned positive for Novel SARS-CoV-2. We initiated contact tracing among his immediate family members to ascertain the level of person to person transmission. Nasopharyngeal and throat samples were taken from six (6) family members and were taken to the National Reference Lab of NCDC in Abuja for analysis. After two days the results retuned positive for four family members within the family unit.

## Results

### Clinical characteristics

**Case 1: familial index case:** on the 1^st^ May, 2020. A patient (case 1) from a family unit presented himself to the Federal Medical Center Keffi, in Nasarawa State, North Central Nigeria with pulmonary infiltrates, respiratory symptoms and fever. His symptoms fit into the case definition guideline of the Nigeria Center for Disease Control (NCDC). Preadmission evaluation also reveals his body temperature of 42°C, full blood count of 11.2 cells per liter and calcification of the aorta and aortic wall with pulmonary hypertension of both lungs. He has negative results for influenza A and B which was negative but had hyperglycemia with fasting blood sugar (FBS) of 23 milligrams per deciliter (mg/dL). Interstitial hyperplasia was reveal by computer tomography with infection in the left lungs posterior upper lobe segment. His throat and nasopharyngeal swab were taken and placed in a viral medium for testing of Novel SARS-CoV-2 by real time PCR. On the 2^nd^ May, 2020 by 10:03 am the patient died and PCR results returned positive for Novel SARS-CoV-2 (Table 1).

**Table 1:** socio-demographic and clinical characteristics of positive familial cluster cases of Novel SARS-CoV-2 in Nasarawa State, North Central, Nigeria

	Case 1	Case 2	Case 3	Case 4
Relationship	Family index case	Wife to index case	Brother to index case	Brother to index
**Age (Years**)	43	39	40	38
**Sex**	Male	Female	Male	Male
**Occupation**	Politician	House Wife	Businessman	Student
**Comorbidities**	Diabetes	None	Chronic Sinusitis	Diabetes
**Signs & Symptoms**				
Difficulty in breathing	+	+	+	+
Fever	+	+	+	+
Cough	+ (Productive)	+ (Dry)	+ (Productive)	+ (Productive)
Generalized weakness	+			
Body temperature (℃)	42.3	39.2	37.6	30.2
Diarrhea	+	+	+	+
Oximetry saturation (%)	96	95	97	98

NA: not available, + positive, - negative

**Case 2:** case 2 is a 39 years old female, a wife to the index case (conjugal relationship). She had a close contact with case 1 as a wife and care-giver before his demise. 6 days after his demise, her throat and nasopharyngeal samples were taken and she was line-listed. She had diabetes and hypertension as underlying clinical conditions with difficulty in breathing, fever, and dry cough with generalized body weakness. Her body temperature was 39°C without diarrhea, with 92% oximetry saturation. Her results returned positive according to Novel SARS-CoV-2 nucleic acid test (Table 1).

**Case 3:** case 3 is a 40 years old male, a business man who is a sibling to case 1. He was among those that conveyed the corpse to the family house, bathed the corpse, and took it to the grave side for the burial right. Few days, he presented with chronic sinusitis with hyperglycemia and difficulty in breathing. Fever, dry cough with general body weakness, and body temperature of 37.6°C with absence of diarrhea and 72% oximetry saturation. Novel SARS-CoV-2 nucleic acid test returned positive. He was isolated and taken to a designated isolation center (Table 1).

**Case 4:** case 4 is a 38 years old male, who is a sibling to the index case. He has diabetes as underlying health condition. He was in company of case 3 that bathed the corpse of case 1. He was line listed and contact traced. Few days, he presented with fever, difficulty in breathing, productive cough, and general body weakness with body temperature of 39.2°C and oximetry saturation of 90% his Novel SARS-CoV-2 nucleic acid test returned positive. He was isolated and taken to an isolation center (Table 1).

## Discussion

In this familial cluster analysis for novel corona virus 2019 infection. Throat and nasopharyngeal swab samples for the 4 cases were tested and confirmed positive for 2019-nCoV according to real time PCR nucleic acid detection protocol. One of them had a radiological changes which support viral pneumonia. Worthy of note, case 1 (the familial index case) had no travel history to any country of high risk, but been a Politician, his association with electorates might have mimic his positive status to COVID-19. Initial source of infection to the Index case was not known. Source of infection to case 2, case 3, and case 4 has been with case 1. This is because they live in the same apartment, case 3 and 4 handled the corpse of the Index case upon demised. This finding indicated that a person with Novel SARS-CoV-2 might be infectious during incubation period. Zeng *et al*. [10]. Report reveals SARS cases are infectious during asymptomatic period and incubation period [11]. Middle East Respiratory Syndrome (MERS) and Novel SARS-CoV-2 infect the epithetical cells of the upper airways. Persons who have contact with Symptomatic patients with SARS-COV-2 should be isolated for further review and treatment.

The throat and nasopharyngeal swab samples of four cases in our studies were positive to SARS-COV-2 indicating that SARS-COV-2 can infect the upper respiratory tract. These results is in agreement of hypothesis that 2019-nCoV can be easily discharged through the respiratory tract [12] One of the biggest challenges is the understanding of the infectivity rate during incubation period of 2019-nCoV, especially with the source of infection and isolation of contacts (close) as new consideration. Nigeria been a unique nation with peculiar commonplace, large social gathering such as burial and wedding ceremonies, peculiar in religion tourism needs to adopt social and physical distancing strategies. Mass gathering such as burial ceremonies like it is seen in our study has been associated with Novel SARS-CoV-2 transmission creating an Eco-system for increase in transmission thereby creating high risk conditions linked to social gathering cluster as seen in Singapore [13]. It´s not surprising to see that this year, countries around the world cancel pilgrimage (Umar) in Saudi-Arabia [14].

## Conclusion

Our study revealed that person to person transmission within family cluster or nosocomial infection is possible in setting where precautions such as personal hygiene, social distancing and the use of personal protective equipment are not adhered to. Clinicians should be aware of clinical history of contact patients to enable them promptly identify in order to curb further spreading in hospital and family cluster. Our recommendation will be for adoption of National Guideline that will reveal epidemiological exposure history as important reference point for identifying source of infection and strengthened, protection and isolation measures. Close contacts to confirm cases should be included highly suspected cases during Incubation period of confirmed cases. Availability of high sensitive rapid diagnostic reagents for Novel SARS-CoV-2 should be accelerated in order to facilitate community testing.

### What is known about this topic

- Person to person transmission of SARS-CoV-2 has been established;
- Similar comorbidities are also associated with risk of severe outcomes with people infected with SARS-CoV-2.

### What this study adds

- Cluster familial analysis reveals community and household transmission;
- Strategies to interfere with transmission circle revealed.
